# War Influences on Medical Staffs and the Profession

**Published:** 1915-10-23

**Authors:** 


### WAR INFLUENCES

### ON MEDICAL STAFFS AND THE PROFESSION.

There are few, if any, of the ordinary civilian professions whose *personnel* have been so directly and so greatly affected by the European war as that of medicine; and there have been few, if any, of the charitable institutions of the country that have felt the same pinch so much as the voluntary hospitals. The mere fact that the latter depend so utterly upon the gratuitous services of their medical staffs is one of the reasons, though not the only one, why anything which profoundly affects the medical profession must affect the hospitals too. Indeed, the problems of carrying on our hospitals in spite of the shortage of medical men, which is already seriously felt—and is likely to be worse before it is better—are only to be solved by taking broad views of the present position in the profession; and perhaps by modifying or abandoning some of the preconceived ideas which in long years of peace had grown into acknowledged and recognised conventions.

Let us first glance briefly at the position in regard to the supply of resident medical officers, house surgeons, house physicians, and the like; that is to say, the most junior and most transitory members of the medical staffs of hospitals. Before the war one could roughly classify hospitals—from the resident medical officer standpoint—into those which obtained their residents from an attached medical school, and those which, having no medical school, took men trained elsewhere. In the main, the former class paid no salaries to these medical officers, but provided merely board, lodging, and sometimes washing; whereas the latter class offered in addition a salary of variable amount. The reason why the "teaching hospitals" paid no salary was that the prestige of holding an appointment there was supposed to be sufficient reward for the services of the young surgeons and physicians who applied for the post. For the most part there was no lack of applicants on those terms; so that by the ordinary laws of supply and demand the hospitals were justified, on business principles, in their attitude. The salaries offered by the second type of hospital were for the most part small, ranging from £50 a year up to £100, rarely more. It is worth noting that in the last decade the figures have been steadily rising; many hospitals now offer pay which formerly did not, and many have had to raise their rates of salary in order to attract applicants.

What is the position now? Any young doctor, brand-new qualified and without any experience whatever, can get nearly £500 a year by joining as a temporary lieutenant in the R.A.M.C., with board and lodging, and the added attraction of a commission and war service. Can it be wondered at that the hospitals have difficulty in getting the men they want? A reference to our advertisement columns shows the steps that are being taken to get the vacancies filled. Salaries up to £150 for even assistant house surgeons are being given by institutions which a few years ago would have offered nothing, or, at the most, £50. Female residents are being appointed at many hospitals where until war broke out none but males needed to apply. The new War Office scheme, by which at approved teaching hospitals young doctors taking house appointments receive honorary commissions, and after three months are liable to be called up for service in the R.A.M.C., is also being taken advantage of: thus the Bristol Royal Infirmary, to quote an instance, is advertising for residents under this scheme, and offers them pay at the rate of £120 a year in addition. Then, again, a certain number of men have taken up house appointments at an age very different from that of the ordinary resident. At one London hospital, for instance, two members of the visiting staff are now living in the hospital and acting as house surgeons; and at another an even stranger sight may be seen, for one of the resident house surgeons is, and has been for years, a member of the visiting staff of another hospital. Probably careful inquiry would show that many such appointments have been made in recent months; and, as most of these surgeons are ineligible for military service, the principle is one for which there can be nothing but approval. Indeed, one or two institutions advertising lately for residents have expressly stated that no candidate eligible for the R.A.M.C. will be appointed. One way and another, then, the voluntary hospitals are managing to eke out in the matter of residents, though precariously and with a good many makeshift arrangements.

### The War and the Visiting Staffs.

From the simple numerical aspect, the war has caused less dislocation among the visiting than among the resident staffs, because the former include a high proportion of men over military age. A fair percentage of the assistant visiting surgeons have gone into the R.A.M.C., and a good many hospitals have had difficulty with their out-patient departments in consequence: a case in point, typical of many others, is the Manchester Infirmary, which has had to close one of its out-patient clinics for want of staff. Here and there a senior surgeon or physician has left to act as consultant to the Army abroad; and at some hospitals, but not a great many, there has been a really serious difficulty in getting the wards properly staffed. But, in the main, such disturbances to the established order of things have been surmounted more or less satisfactorily without any great difficulty by the ungrudging patriotism with which the work of absentees has been shouldered by those whom duty has not called away.

The foregoing applies to the mass of the voluntary hospitals, big and little. But there are certain important exceptions where the war is more vitally felt and appreciated—namely, in those hospitals whose staffs were *à la suite* officers of the Territorial General Hospitals before the war, and were called up for duty after the mobilisation. These hospitals include most of the best-known teaching hospitals, and there is a good deal of variety in the exact extent to which they are affected. At some hospitals part of the regular wards have been placed at the disposal of the War Office for the reception of soldiers; extra improvised beds have been added thereto in some cases. In such cases the regular hospital staff does the work of the hospital as usual, though military surgery is the order of the day instead of the profoundly different science and art of peace surgery. At other hospitals, and this is the usual procedure, the actual general hospital is housed in buildings quite separate and distinct from the civilian hospital, which continues its ordinary avocation of healing the civilian population. The military hospital has the first call upon the services of the staff, who can attend their civilian wards afterwards. In practice a scheme is often adopted by which half the staff do purely civilian and half purely military duties for three months at a time, and then change over for the next three. This plan seems to work well, and is probably the most economical and efficient in the long run for everybody.

### The War and Private Practice.

On the medical profession in general the influence of the war has been far-reaching indeed; nor is it possible to envisage it quite so summarily as that upon the staffs of hospitals. To begin with, there is a consensus of opinion that private practice—excluding all forms of contract practice—has decreased considerably in volume; indeed, those who practise chiefly among the middle and upper classes declare that it has fallen by 50 per cent. If that is so, the probable explanations are not that the population is healthier, but that the *malade imaginaire* class has too many other things to think about to send for a doctor, and that everyone is economising as much as possible. Among the consultants there is not quite unanimity; some declare they are doing as much as before the war, others bewail an enormous drop in their *clientèle*. Certainly there is a tendency towards a lowering of fees, which doubtless denote a lessened total demand for their services. In other words, all classes of medical men at home, save those who depend chiefly upon panel and club practice, are finding their incomes seriously reduced by the war. Another very serious matter, financially, for considerable numbers in the profession is the amount of work for wounded soldiers which is expected gratuitously. Many surgeons are complaining that these calls upon their energies are becoming too great.

There are, however, large sections of the profession to whom this does not apply. As has been already mentioned, young graduates can get nearly £500 a year by joining the R.A.M.C. or the Navy, and nearly as much from the Red Cross Society. Needless to say this is very greatly above the average income to which such young doctors can aspire in the usual way. Then, again, there are professional *locum tenentes*, many of whom are unfit or ineligible for military service. They are reaping a harvest, indeed, for they are commanding salaries such as have never been given before; and the supply is nowhere near equal to the demand.

Many thousands of practitioners are serving in the R.A.M.C. or in the Navy Medical Service. Some of the former belong to the Special Reserve, more to the Territorial Force, and more still are temporarily enlisted men. Many of these doctors have left their homes and practices to serve their country's need, and many of them are sacrificing not only present income but future prospects as well in so doing. The younger ones are doing well for themselves; but most of the others are losing money, except a few of those who by dint of many years' unremunerated services in the Territorials had acquired sufficient seniority to rank and draw pay as majors and lieutenant-colonels.

What the effects of the war upon the future prospects of the profession will turn out to be is a question that is much discussed by the R.A.M.C. abroad. The general impression is that many of the young men who are crowding into the temporarily commissioned R.A.M.C. and into the Special Reserve will never settle down afterwards into the humdrum life of private practice. Probably more of them will do so than is generally believed, though no doubt a good many will not. Then, again, a certain number have been killed or disabled; not a high proportion, but enough to make a distinct difference if there really should be a shortage of qualified men after the war. There seems, further, to lie ahead of us a period of some few years in which a diminished annual entry into the profession is assured on account of so many junior medical students entering the Army as combatants. The result of this upon the future of those already qualified and practising is the subject of a good deal of optimistic speculation, the fulfilment of which remains to be seen.

